# Local multifrequency impedance changes after radiofrequency ablation in human atria: potential use for tissue characterization

**DOI:** 10.3389/fcvm.2025.1668533

**Published:** 2025-10-07

**Authors:** Gerard Amorós-Figueras, Zoraida Moreno-Weidmann, Francisco Méndez-Zurita, Marc Soriano-Amores, Ramon Bragós, Javier Rosell-Ferrer, Jose M. Guerra

**Affiliations:** ^1^ Department of Cardiology, Hospital de la Santa Creu I Sant Pau, Institut de Recerca Sant Pau (IR SANT PAU), CIBERCV, Universitat Autònoma de Barcelona, Barcelona, Spain; ^2^ Electronic and Biomedical Instrumentation Group, Department of Electronics Engineering, Universitat Politècnica de Catalunya, Barcelona, Spain

**Keywords:** local multifrequency impedance, radiofrequency ablation, electrophysiology, tissue characterization, lesion assessment

## Abstract

**Background:**

Local impedance (LI) mapping provides additional tissue characterization of the atria substrate. Measuring LI at different current frequencies has the advantage of exploring intra- and extra-cellular compartments and may add useful information about tissue integrity. The objective of this study was to characterize the changes in local multifrequency impedance (LMI) after radiofrequency ablation in human atrial tissue.

**Methods:**

In fifteen patients undergoing catheter ablation of atrial arrhythmias, we constructed a baseline high-density electroanatomical map (EAM) and measured the LMI (1–1,000 kHz) at fifty sites around the cava veins using the QDOT or Smarttouch electrocatheter. Then a point-by-point pulmonary vein isolation procedure was performed using radiofrequency energy in a temperature controlled mode (90W for 4 s for QDOT/30W for 30 s for Smarttouch). After confirming the PVI fifty additional LMI recordings per patient were performed around the initial sites. We performed an offline analysis to compare the values of bipolar voltage and LMI of blood, pre- and post-ablated tissue. We also analyzed the cardiac cycle changes of LMI and the effects of catheter orientation to the LMI, contact force and bipolar voltage.

**Results:**

A total of 641 pre-ablated and 190 post-ablated sites were studied from all patients. Blood pool, healthy and post-ablated myocardium presented distinctive LMI signatures (Z_PRE_ = 110 ± 15 Ω vs. Z_POST_ = 90 ± 10 Ω vs. Z_BLOOD_ = 90 ± 8 Ω; *p* < 0.001). LMI cyclic changes showed an inverse relationship with the contact force, and these were more attenuated in the post-ablated tissue (*p* < 0.001).

**Conclusions:**

LMI can differentiate pre- from post-ablated tissue in a cohort of patients submitted to RF ablations. This new tool could be of potential clinical applicability for the characterization of the atrial substrate and to monitor lesion quality to perform durable ablation lesions.

**Clinical Trial Registration:**

NCT05159180 (https://www.clinicaltrials.gov); Unique Protocol ID: IIBSP-IMS-2021-74.

## 1 Introduction

Radiofrequency ablation is an established therapy for atrial fibrillation treatment (1). However, long-term success rates remain suboptimal, with recurrence rates around 30% (2). This can be partially attributed to the limitations of the current mapping systems to characterize the atrial substrate and at the same time predict the lesion efficacy during the radiofrequency (RF) ablation procedures. The algorithms of those systems to characterize the atrial substrate are based on passive tissue properties such as local voltage. The lesion quality indicators have been developed in experimental models targeting healthy myocardium and are based on external biophysical properties such as power, temperature, duration of RF and contact force (3). In contrast, the generator impedance (GI) measured by the commercial RF generators is both a marker of the local biophysical derangements induced in the ablation site and have also been correlated with the ablation lesion size in experimental studies (4, 5). Recent clinical studies have also shown that local impedance, measured at the catheter tip, is more effective than GI and can be used to characterize myocardial tissue heterogeneity (6–10). The measurement of LI at different current frequencies has the advantage of exploring the integrity of intra- and extra-cellular compartments and, in theory, would allow for a better characterization of the ablated and non-ablated atrial myocardial tissue (9, 11). The goal of this study was to characterize the changes of local multifrequency impedance (LMI) occurred in the myocardial tissue before and after radiofrequency ablations in patients submitted to a PVI procedure.

## 2 Methods

### 2.1 Study population

We conducted a single-center, prospective, observational study in fifteen patients (13% females) with atrial arrythmias undergoing catheter ablation. All patients were included from December 2021 to June 2022 and the inclusion criteria were: (1) Age of comprised between 18 and 85 years old, (2) Understanding of the informed consent, (3) That they did not present any contraindication and pass the exploration and tests prior to bioimpedance measurements. The exclusion criteria were: (1) Age outside the range described in the inclusion criteria, (2) Subjects who presented any type of complication during the procedure, (3) Pregnancy. The protocol was approved by the ethics committee and the study was conducted in accordance with the principles of the Declaration of Helsinki. Written informed consent was obtained from all patients prior to study participation.

### 2.2 Experimental procedures in patients

#### 2.2.1 Baseline electroanatomical map

Patients were submitted to mapping of both voltage and local multifrequency impedance. Briefly, patients were anesthetized with intravenous propofol (2–4 mg/kg) and were maintained with a mixture of oxygen and sevoflurane inhalation (2.5%–3.5%). A femoral vein was catheterized and a mapping electrocatheter (Pentaarray, Biosense Webster, USA) was advanced to the left atrium through a transeptal access using fluoroscopic guidance. Then, a 3D high density endocardial mapping of left atria was constructed.

#### 2.2.2 Local multifrequency impedance

**Figure 1:**
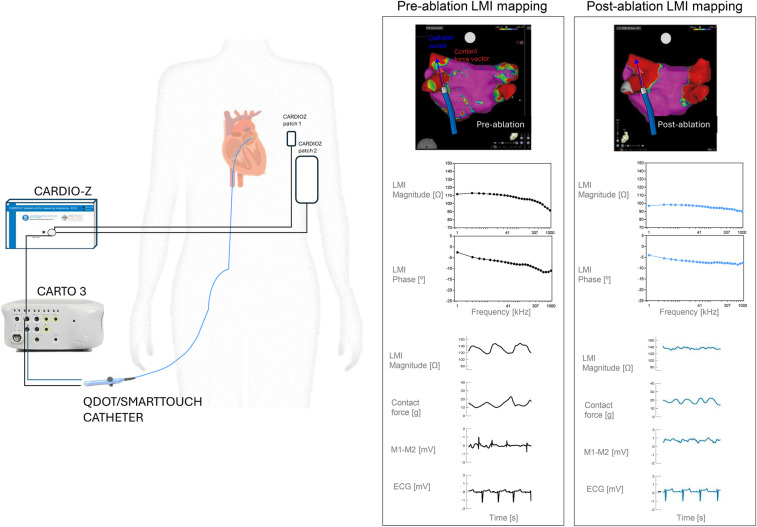
Experimental setup. Left panel shows a basic diagram of the experimental setup used to measure the Local Multifrequency Impedance (LMI) on patients submitted to pulmonary isolation procedure. Right panel shows an electroanatomical map (EAM) of the left atria of a representative patient with its corresponding LMI data, for a selected site.

Afterwards, the mapping catheter was replaced by an irrigated ablation catheter (QDOT or Smarttouch, Biosense Webster, USA) with the aim to measure the local multifrequency impedance. LMI of the left atrium was measured at fifty sites around the cava veins and other anatomical sites using the same endocardial mapping electrocatheter. To ensure precise spatial correspondence between pre- and post-ablation measurements we used manual CARTO point tagging with a minimum contact force for data inclusion of 5 g. This catheter was connected to a CARTO 3, a SmartAblate system (Biosense Webster, USA) and an impedance spectroscopy recording system made by our group (CARDIOZ, Spain) (12). Alternating currents (1 ms duration, 1 mA total peak amplitude) of twenty-six frequencies ranging from 1 to 1,000 kHz were injected between the distal electrocatheter pole and a skin reference electrode (Dispersive pad, 3M) placed before on the anterior thoracic region. The resultant changes in current voltage were measured between the distal electrocatheter electrode and a second thoracic skin reference electrode (ECG pad, 3M, USA) (Figure 1). The LMI was measured at a sampling rate of 60 Hz during the entire duration of the cardiac cycle and stored at 2 s frames. The LMI obtained had two components: (1) the LMI magnitude which quantifies the drop of voltage amplitude for a given applied current and (2) the LMI phase angle that reflects the delay between the voltage and current waves which is influenced by structural characteristics of the myocardial tissue. From all LMI measurement we obtained a frequency-specific response, with lower frequencies reflecting extracellular changes and higher frequencies correlating with membrane integrity (13).

#### 2.2.3 Ablation procedure and parameters

In eleven patients we performed a point-by-point pulmonary vein isolation (PVI) procedure using an irrigated ablation catheter (QDOT, Biosense Webster, USA) with a temperature-controlled mode and a high-power short-duration strategy (90 watts for 4 s). In the other four patients we performed a PVI procedure using another irrigated catheter (SmartTouch, Biosense Webster, USA) with a temperature-controlled mode and a power setting of 30 watts for 30 s. In all patients the efficacy of the ablation was assessed by the reduction of the local electrogram signals. After confirming the PVI fifty additional LMI recordings per patient were performed around the same initial sites.

### 2.3 Signal processing and data analysis

An ofﬂine analysis of the bipolar electrograms (EGM) and of the impedance signals was done using custom-made Matlab scripts (Mathworks, USA) and the following parameters extracted: Bipolar amplitude (in mV) of the EGMs, contact force data, the magnitude and phase angle of the local multifrequency impedance at all current frequencies (in ohms), the amplitude of the cyclic changes of the impedance magnitude at 5 kHz (in ohms), and the impedance magnitude and phase angle drops at all current frequencies (in ohms and percentage). We also analyzed the effects of catheter orientation on the studied variables. For this purpose, we considered that the catheter was orthogonal to the tissue when the angle (ϴ) between the catheter and the contact force was ϴ<|15°|, and that the catheter was parallel to the tissue when the angle between the catheter and the contact force was ϴ>|70°| and ϴ<|110°|.

### 2.4 Statistical analysis

Quantitative data were expressed as the mean ± standard deviation (SD). The ordinary two-way ANOVA test with Sidak's multiple comparison correction was used to assess the statistical significance of changes in bipolar voltages, impedance magnitude and phase angle at different current frequencies (factor 1: blood, pre- or post-ablation; factor 2: current frequencies/catheter used/parallel or orthogonal condition). A *p*-value <0.05 was considered significant. All analyses were performed using SPSS v.22.0 software (IBM-SPSS, USA).

## 3 Results

All patients were included in the study, and a table of their baseline characteristics is shown in Table 1. We obtained high-density electroanatomical maps of all patients. We extracted and merged the data from the CARTO and CARDIOZ systems and obtained a total number of 29 measurements of the blood pool, 641 of the pre-ablated myocardium and 190 of the post-ablated myocardium.

**Table 1:** Baseline characteristics of the study population.

Variable	Mean ± SD/Percentage
Age (years)	55 ± 11
Male sex (%)	87
BMI (kg/m^2^)	29 ± 6
Type of atrial fibrillation:	
Paroxysmal AF (%)	43
Persistent AF (%)	57
Left atrial diameter (mm)	41 ± 4
Left ventricular ejection fraction (%)	60 ± 6
Duration of AF (years)	3 ± 2
Hypertension (%)	36
Diabetes mellitus type 2(%)	21
Dyslipidemia	43
Smoker (%)	29
CHA2DS2-VASc score	1 ± 1

### 3.1 LMI of blood pool, pre-ablated and post-ablated myocardium in patients

**Figure 2:**
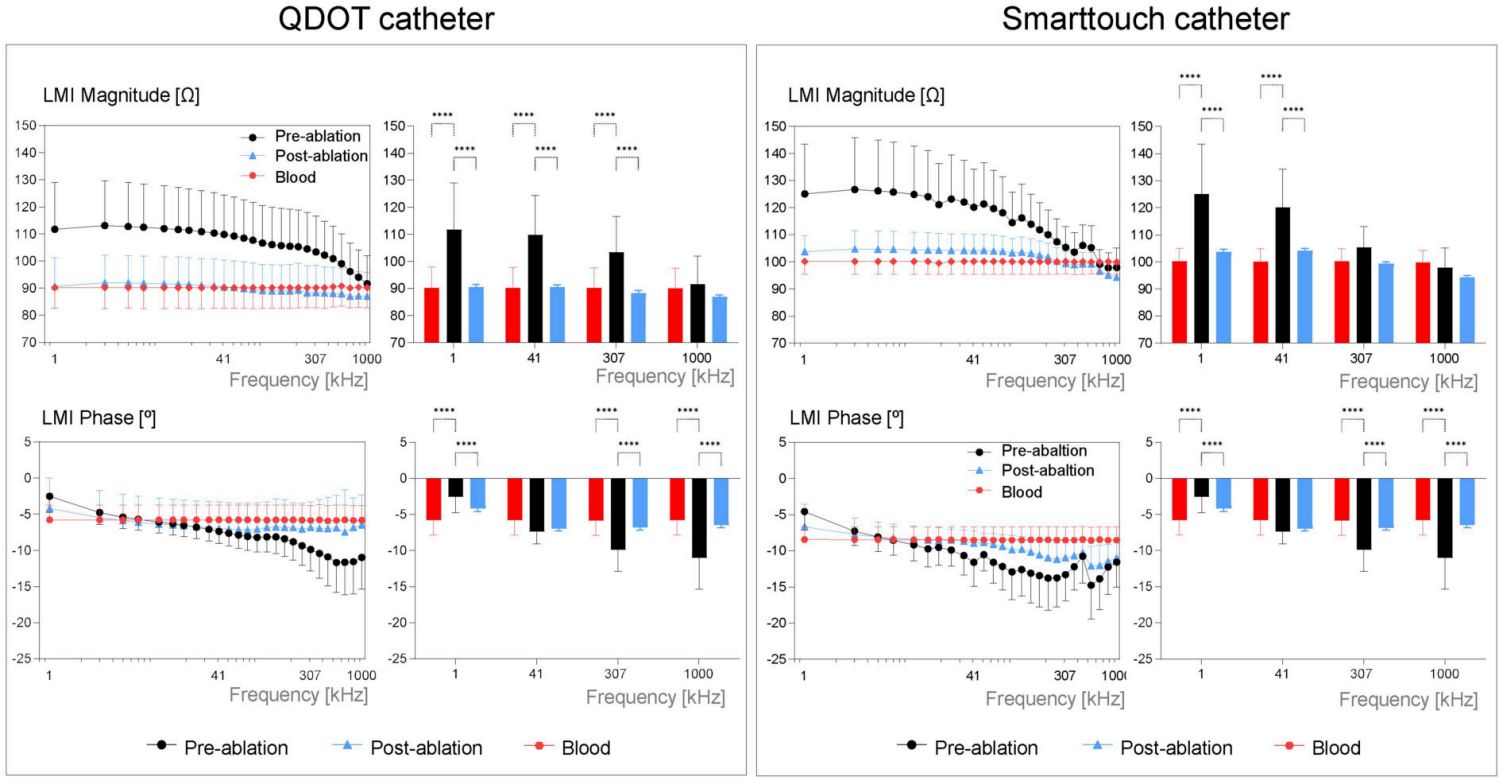
Local multifrequency impedance of pre-ablated, post-ablated and of blood for the QDOT catheter (left panel) and the smarttouch catheter (right panel). Bar graphs show the mean LMI magnitudes and phase angles at selected frequencies for each catheter, in the studied sites. Values are reported as mean ± SD. *** *p* < 0.001, **** *p* < 0.0001.

The LMI magnitude of pre-ablated myocardium was significantly higher than of post-ablated myocardium and of blood pool, from 1 kHz to 209 kHz, in both catheters (ANOVA *p* < 0.05). LMI magnitude of blood pool and post-ablated did not show differences. The LMI phase angle showed similar results (Figure 2). All LMI magnitude values acquired with the QDOT catheter were lower than the measurements performed with the SmartTouch catheter (ANOVA *p* < 0.001). However, the LMI drop was similar among both catheters. The current frequency range that better discriminated pre-ablated from post-ablated regions was 1–371 kHz for the LMI magnitude and 173–1,000 kHz for the LMI phase angle. Detailed impedance spectra for each patient are shown in Supplementary Figure S1.

### 3.2 Cyclic changes of LMI, contact force and local electrograms in patients

**Figure 3:**
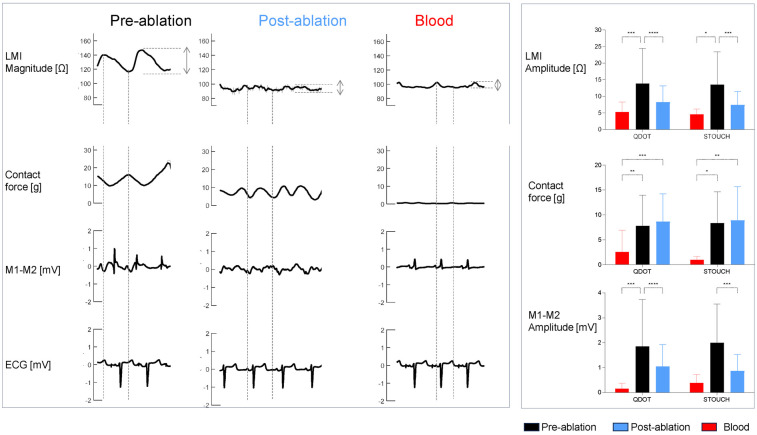
LMI magnitude at 5 kHz, contact force, bipolar electrograms and ECGs (V1) waveforms of one patient recorded in pre-ablated, post-ablated and blood sites (left panel). The right panel shows the bar graphs of the mean amplitudes for the LMI magnitude and the local electrograms and the mean values of the contact force, for each catheter in the studied sites. Values are reported as mean ± SD. * *p* < 0.05, ** *p* < 0.01, *** *p* < 0.001, **** *p* < 0.0001.

Figure 3 illustrates the biphasic pattern of the LMI magnitude during the cardiac cycle and its relationship with the contact force, the bipolar electrograms and the surface ECG. The pre-ablated myocardium had an LMI amplitude higher than the post-ablated myocardium and the blood pool from 1 to 95 kHz, in both catheters (LMI magnitude amplitude using QDOT @ 41 kHz: Z_PRE_ = 12 ± 8 Ω vs. Z_POST_ = 7 ± 4 Ω; ANOVA *p* < 0.001). There were no statistical differences between post-ablated tissue and blood, despite the catheter being used. As expected, the mean amplitude of the bipolar electrograms was higher in the pre-ablated sites than in the post-ablated sites (M1-M2 amplitude using STOUCH: V_PRE_ = 2 ± 2 mV vs. V_POST_ = 0.9 ± 0.7 mV; ANOVA *p* < 0.05). The mean contact force recorded was comparable between the pre- and post-ablated sites and was higher than in blood pool for both catheters (Contact force measured using STOUCH: F_PRE_ = 8 ± 6 g and F_POST_ = 9 ± 7 g vs. F_BLOOD_ = 1 ± 1 g; in both, ANOVA *p* < 0.05).

### 3.3 Influence of catheter orientation in the EGM and LMI measurements in patients

**Figure 4:**
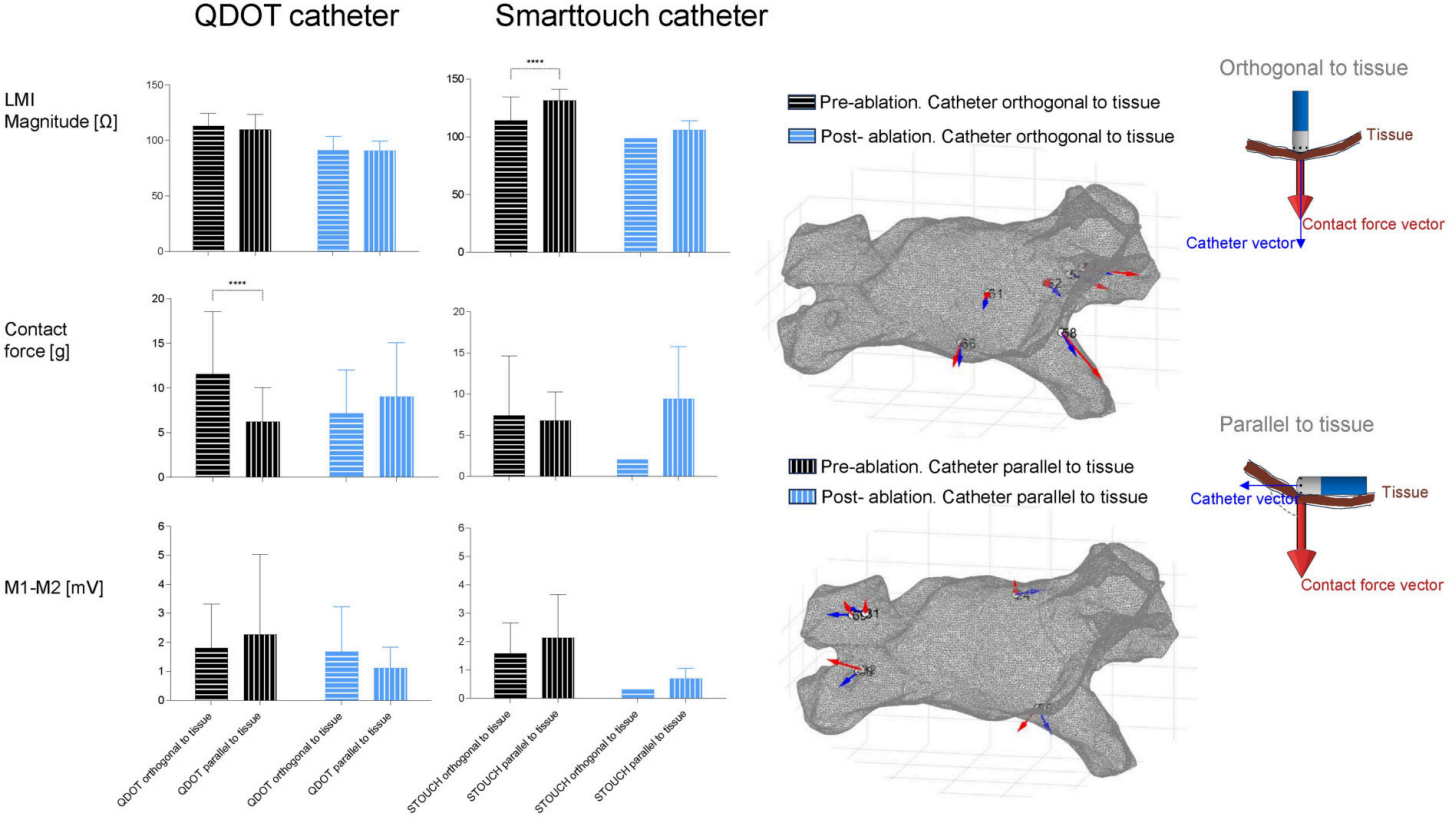
Influence of catheter direction on studied variables. The left part shows the mean values of the LMI magnitude at 5 kHz, contact force and bipolar electrograms in the pre- and post-ablation groups and how the catheter is oriented. The right part shows two geometries of the atria of one patient with different measured sites. In each site the vectors of the catheter direction (blue arrows) and the contact force (red arrows) are represented.

In a subset of the explored sites, we analyzed the influence of catheter orientation on LMI, the contact force and bipolar electrograms. In Figure 4 we can observe that LMI Magnitude changed only in pre-ablated myocardium when using the SmartTouch catheter (LMI measured using STOUCH @ 5 kHz Orthogonal vs. Parallel: 114 ± 21 Ω vs. 131 ± 10 Ω; ANOVA *p* < 0.001). The contact force was higher in the pre-ablated sites only when using the QDOT catheter if it was orthogonal to the tissue. Local bipolar electrograms showed the same behavior despite catheter orientation.

## 4 Discussion

### 4.1 Main findings

This study shows for the first time that local multifrequency impedance can be used to distinguish pre-ablated from post-ablated atrial myocardial tissue, in patients submitted to radiofrequency ablations in PVI procedures.

### 4.2 Ability of myocardial impedance to identify ablated tissue

A fifteen to twenty ohms drop in local impedance during RF ablation is associated with successful lesion creation, as shown in Table 2. This is in accordance with our mean impedance magnitude drop at the current frequency used by commercial RF generators (50 kHz).

**Table 2:** Recent clinical studies on local impedance and RF ablation.

Study type	# of patients	Ablation type	Catheter	LI drop predicts conduction block	Ref
Retrospective, single center, observational	50	CTI	IntellaNav MiFi	>15 Ω	(14)
Prospective, single center, pilot study	8	PVI	StablePoint	>22 Ω for anterior wall >18 Ω for posterior wall	(15)
Prospective, multicenter trial	60	PVI	IntellaNav MiFi	>16 Ω for anterior wall, >12 Ω for posterior wall	(16)
Prospective, multicenter trial	51	PVI	IntellaNav MiFi	>17 Ω for anterior wall >14 Ω for posterior wall	(16)
Prospective, multicenter trial	324	PVI	StablePoint	>21 for anterior wall, >18 Ω for posterior wall	(17, 18)

Recent studies have shown that the optimal LI drop for effective lesion formation depends on the atrial region where the lesions are performed. Higher cut-off values are needed for the anterior wall which has higher wall thickness compared to the lower cut-off values needed on the thinner posterior wall (10, 16). In addition the baseline LI and the LI drop differ according to atrial tissue characteristics: normal-voltage areas show higher baseline LI and greater LI drops, suggesting that tissue substrate should be considered for tailored ablation strategies (9, 17, 19). In this sense, the measurement of impedance at different frequencies allows a better tissue characterization than LI since it permits the detailed characterization of the intra- and extracellular compartments (13). In our study we have described for the first time that human blood pool and post-ablated tissue have a lower LMI magnitude and phase angle than pre-ablated tissue, at all current frequencies studied. The lack of significant differences between post-ablated scarred tissue and blood pool suggests that LMI drops reflect cellular disruption and extracellular matrix disorganization post-ablation, consistent with irreversible lesion formation. This in accordance with previous large-animal studies where the impedance magnitude and phase angle showed the same behavior in the atrial and ventricular scarred tissue (9, 11). The frequency-specific responses, with lower frequencies reflecting extracellular changes and higher frequencies correlating with membrane integrity, further highlight LMI's potential for evaluating the tissue substrate characteristics to perform tailored applications and at the same time evaluate in real-time the lesion formation.

Finally, this study also shows that, despite the ablation power strategy used, 90W for 4 s (QDOT) or 30W for 30 s (Smarttouch), there is a similar LMI drop. This is in accordance with a recent experimental study where different high power short duration strategies and catheters were compared and showed similar impedance drops (20).

### 4.3 Cyclic changes of LMI

The biphasic pattern of LMI magnitude during the cardiac cycle highlights its dynamic interaction with mechanical and electrical activity (21, 22). Pre-ablated myocardium exhibited 171% greater cyclic impedance amplitude than post-ablated tissue, reflecting preserved tissue elasticity and contact force variability. The time-correlation between LMI fluctuations and contact force emphasizes the importance of stable catheter contact during ablation. Post-ablated tissue's attenuated cyclic changes may indicate loss of structural integrity, serving as a functional marker of lesion maturity. These findings suggest that integrating both static multifrequency impedance values and dynamic cyclic behavior could enhance lesion assessment.

### 4.4 Influence of catheter orientation

Catheter orientation significantly impacted LMI measurements in pre-ablated tissue when using the SmartTouch catheter, but it did not have an effect when using the QDOT catheter. This discrepancy may stem from differences in electrode configuration or contact force sensing mechanisms between catheters. Bipolar electrogram voltages remained unaffected by orientation. Both results are in accordance with a recent *in vitro* study by Calzolari et al. where they found that changes in catheter orientation did not affect the lesion depth (23).

### 4.5 Limitations

This study has several limitations. First, the sample size was modest, and the single-center design may limit generalizability. Second, differences in LMI values between catheters (QDOT vs. SmartTouch) complicate direct comparisons, requiring catheter-specific validation. Third, the fact that the impedance signature of a successful lesion converges with that of blood, potentially limits the specificity of the impedance measurement for distinguishing an effective ablation lesion from the pool of blood near the catheter tip. However, the integration of the magnitude and phase angle together with the cyclic changes of LMI and the contact-force data could overcome this issue. Finally, while LMI's cyclic behavior was characterized, its clinical utility for predicting lesion durability remains speculative without further validation using imaging methods.

### 4.6 Clinical implications

This study shows that measuring local impedance at different current frequencies is feasible in the clinical scenario. The integration of LMI with electroanatomic mapping systems may enable detailed local tissue characterization and at the same time automated lesion tagging, optimizing ablation line continuity. Furthermore, cyclic impedance patterns could serve as intraprocedural biomarkers of contact stability and lesion transmurality. Recent studies have shown that the deepest penetrations can be achieved using an ablation strategy consisting of applying 50W for 10–15 s (24). Future studies should explore LMI-guided ablation strategies using this power setting and others to determine their impact on long-term arrhythmia-free survival.

### 4.7 Conclusions

This study shows for the first time that LMI can differentiate pre- from post-ablated tissue in a cohort of patients submitted to RF ablations. By combining frequency-specific impedance measurements with dynamic cyclic analysis, this new tool could be of potential clinical applicability for the characterization of the atrial substrate and at the same time monitor lesion quality during the ablations.

## Data Availability

The data underlying this research will be publicly shared on reasonable request to the corresponding author.

## Supplementary material

Supplementary Figure 1 Local multifrequency impedance of pre-ablated, post-ablated and of blood for the QDOT catheter (left panel) and the Smarttouch catheter (right panel) for all the patients of the study.
