# Supplementary figures and images for: Local multifrequency impedance changes after radiofrequency ablation in human atria: potential use for tissue characterization
Source: Front Cardiovasc Med. 2025 Oct 7;12:1668533. doi: 10.3389/fcvm.2025.1668533 (PMC12537893; doi:10.3389/fcvm.2025.1668533)

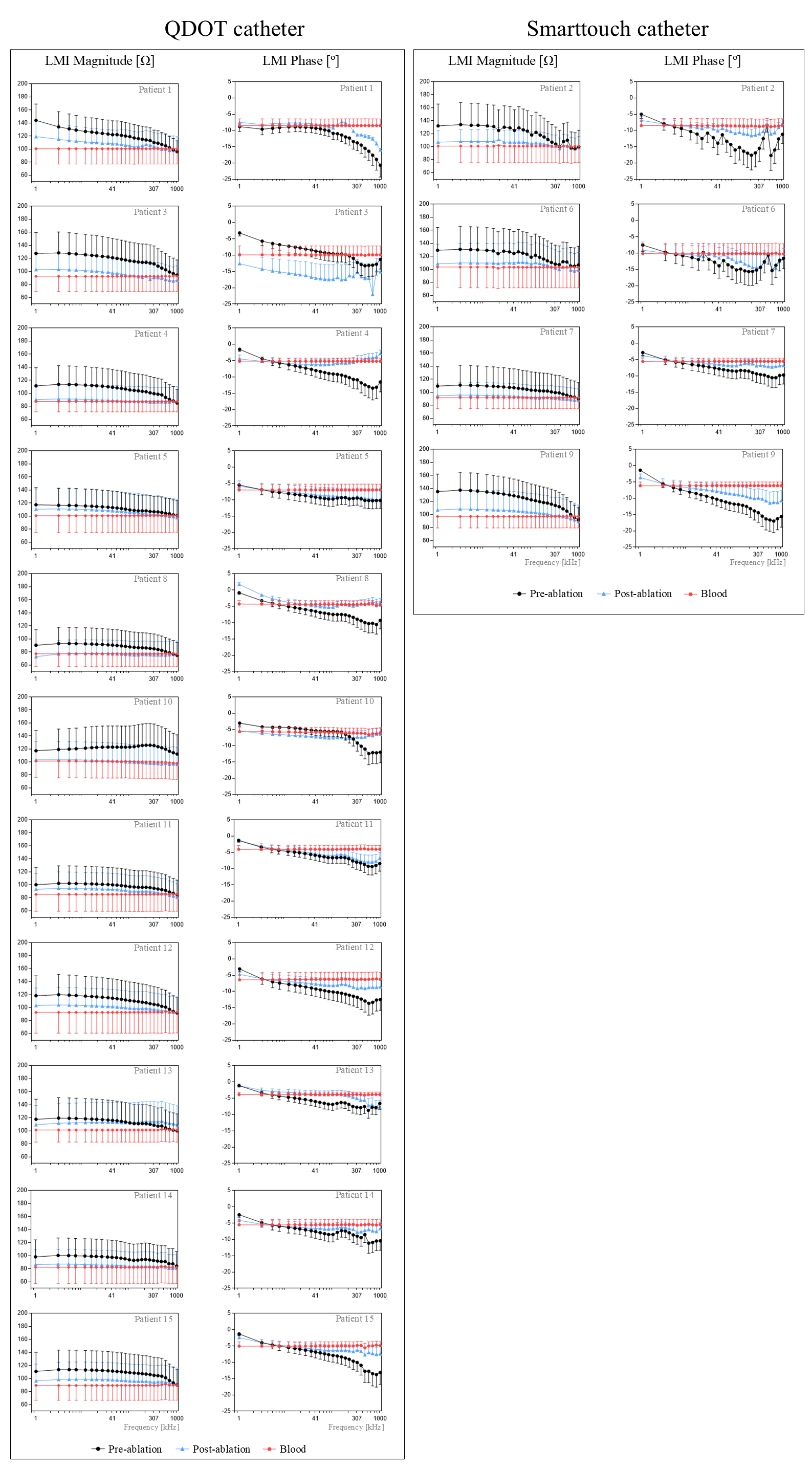

Supplement: Supplementary Figure 1 — Local multifrequency impedance of pre-ablated, post-ablated and of blood for the QDOT catheter (left panel) and the Smarttouch catheter (right panel) for all the patients of the study. [file Image1.tif]
